# Health system readiness to manage maternal death data and avail evidence for decision-making through the Maternal Death Surveillance System in Ethiopia, 2020

**DOI:** 10.1186/s12913-023-09321-x

**Published:** 2023-03-31

**Authors:** Abduilhafiz A. Endris, Tizita Tilahun

**Affiliations:** 1 Ethiopian Public Health Institutes (EPHI), Public Health Emergency Management (PHEM) Center, Maternal Death Surveillance and Response (MDSR), Addis Ababa, Ethiopia; 2 College of Health Science, Family Health and Population Department, Bahirdar University, Bahirdar, Ethiopia

**Keywords:** Health system readiness, MDSR, System evaluation, Ethiopia

## Abstract

**Background:**

Maternal mortality remains a major health problem in Ethiopia. To generate contextual evidence on the burden and distribution of existing causes and contributing factors for programmatic and individual-level decision-making, the Maternal Death Surveillance and Response System was introduced in 2013. This assessment describes the Ethiopian health system's readiness to avail evidence for decision-making through the MDSR system.

**Method:**

A cross-sectional study designed using the WHO framework for evaluating surveillance systems was used. By employing a multistage sampling, 631 health facilities and 539 health posts were included. ODK collect data entry software was used to collect data from September 2019 to April 2020. Findings are presented in text descriptions, graphs, maps, and tables.

**Findings:**

Four hundred (77.1%) health facilities (332 (74.6%) health centers and 68 (91.9%) hospitals) and 264 (71.5%) health posts reported implementing the MDSR system. Of the implementing health facilities, 349 (87.3%) had a death review committee, and only 42 (12.4%) were functional. About 89.4% of health centers and 79.4% of hospitals had sub-optimal maternal death identification and notification readiness. Only 23 (6.96%) and 18 (26.47%) MDSR-implementing health centers and hospitals had optimal readiness to investigate and review maternal deaths, respectively. Moreover, only 39 (14.0%) health posts had locally translated case definitions and 28 (10.6%) had verbal autopsy format to investigate maternal deaths. Six (1.5%) facility officers and 24 (9.1%) health extension workers were engaged in data analysis and evidence generation at least once during 2019/20. Regional variation is observed in system implementation.

**Conclusions and recommendations:**

Sub-optimal MDSR system implementation is recorded. Revitalizing the system by addressing all system components is critical. Having a national-level roadmap for MDSR system implementation and mobilizing all available resources and stakeholders to facilitate this is vital. Establishing a system for routine data quality monitoring and assurance by integrating with the existing PHEM structure, having a system for routine capacity building, advocacy, and monitoring and evaluating the availability and functionality of MDSR committees at health facilities are all critical. Digitalization, designing a system to fit emerging regions' health service delivery, and availing required resources for the system is key.

## Background

### Introduction

Globally, maternal mortality continues to be a significant public health concern. In 2017, maternal mortality ranged from 462 per 100 000 live births in the least-developed countries to 11 per 100 000 in high-income countries. The report also indicated that low and middle-income countries in Sub-Saharan Africa and Southern Asia make up 86% of global maternal deaths [1, 2]. Based on the Sustainable Development Goals (SDGs) goal for 2030, all countries should reduce MMR by two-thirds of their 2010 baseline level. The average global target is an MMR of less than 70/100 000 live births by 2030. However, accurate information on how many women died, where they died, and why they died is essential yet currently inadequate [3].

Ethiopia has one of the highest Maternal Mortality Rates (MMR) globally, with an estimated maternal mortality ratio of 412 maternal deaths per 100,000 live births [4]. On the path to ending preventable maternal mortality, the WHO guide "Beyond the numbers" was launched in 2004 to encourage not just estimating the number of maternal deaths to know the magnitude of the problem but also to understanding why and where the women died to be able to do something about it [5–10].

Following the WHO recommendation, the Ministry of Health (MOH) – in Ethiopia established the Maternal Death Review and Response (MDSR), a system to count and investigate all maternal deaths to generate and avail real-time information for decision-making in 2013. Since 2014, the MDSR has been integrated with the national Public Health Emergency Management (PHEM) system. The MDSR system is expected to avail reliable and quality data and information in real-time on the burden, causes, and contributing factors of maternal deaths and preventability of fatalities in the nation, thereby assisting with timely responses for improving maternal in Ethiopia [11, 12]. To date, several promising achievements have been gained. However, one of the challenges in eliminating preventable maternal death in Ethiopia is the absence of information that shows the magnitude and causes of maternal deaths to assist with decision-making regarding the responses [8, 9]. Therefore, this study was conducted to determine the MDSR system implementation status and describe the health system's readiness for generating and availing good quality data and evidence for the decision-making process in Ethiopia.

### Scope of the evaluation and evaluation questions

**Fig. 1:**
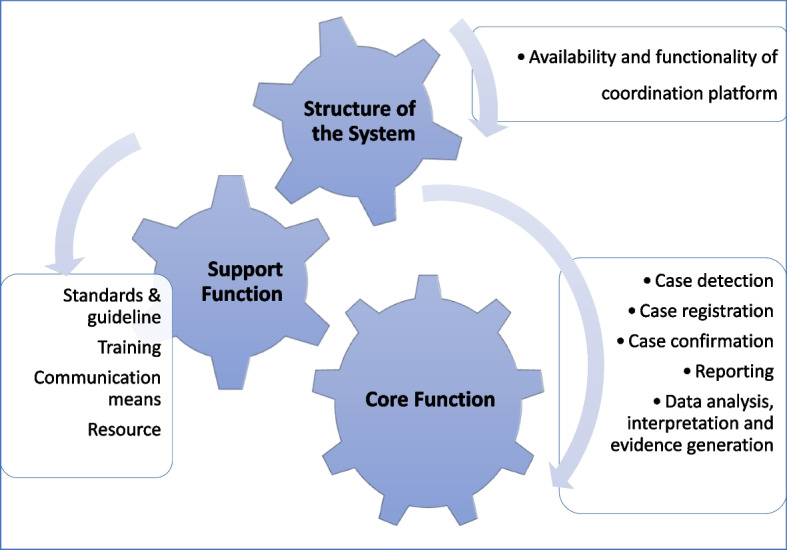
Scope of the national maternal death surveillance and response evaluation, national MSDSR system evaluation, Ethiopia

This national MDSR system evaluation covers the three components of the Maternal Death Surveillance and Response system in Ethiopia's community and health facility health system structure. The scope of this national MDSR system evaluation is shown below (Fig. 1).

This system evaluation addresses the below-indicated evaluation questions.

- To what extent does the MDSR system structure exist and function at health facilities and in the community?
- Does the national MDSR system have sufficient supporting functions for detecting, notifying, reporting, investigating, reviewing, and generating evidence?
- To what extent can the national MDSR system support maternal death detection, registration, reporting, investigation, review, analysis, and provision of evidence for relevant actors?

## Methods

### Study setting and period

Ethiopia is the second-most populous nation in Africa, with more than 110 million people (CSA 2015), located in the Horn of Africa. A household's average size is 4.6 people. The population's pyramidal age structure has remained predominantly youthful [10, 13]. The total fertility rate is 4.6 children per woman (2.3 in urban areas and 5.2 in rural areas). The Maternal Mortality Ratio is estimated at 412 per 100,000 live births [3].

According to the health sector transformation plan (HSTP), Ethiopian health services are being restructured into a three-tier system; primary, secondary, and tertiary level of care. The primary level of care includes a primary hospital, health center, and health post. The Primary Health Care Unit comprises a health center (HC) and five satellite health posts (HPs). Availability, accessibility, equity, efficiency, and quality of health services depend on the infrastructure's distribution, functionality, and quality [14]. This evaluation was conducted at the national level and included nine regional states of the nation: Tigray, Afar, Amhara, Oromia, Somali, Benishangul Gumuz, the Southern People's Nations and Nationalities (SNNP), Gambella, and Harari, as well as two city administrations: Addis Ababa and Dire Dawa. This evaluation was conducted from September 2019 to April 2020.

### Study design

A cross-sectional study assessed selected health facilities (hospitals and health centers) and health posts in all regions and city administrations. A modified version of the WHO framework for evaluating communicable disease surveillance systems and monitoring and evaluation framework for the MDSR system for Ethiopia were used to design and evaluate the system [12, 15].

### Data sources and survey participants

- Facility-level information and information from experts working in the MDSR system at health centers, hospitals and health posts.

### Inclusion and exclusion criteria

#### Inclusion criteria:

- Public health centers, hospitals, and health posts

#### Exclusion criteria:

- Private health facilities and special clinics focused on other than maternal health, e.g., dental, eye clinic, etc.
- Public health facilities and health posts that were operational in the last fiscal year

### Sample size determination and sampling technique

This study considers the clustering effect units assuming the hierarchical nature of study units. All regions were selected as primary sampling units. Then, the required number of health centers and hospitals was determined at each stratum using stratified random sampling techniques using the types of facilities as stratifying variables. The sample size for this study based on the parameters is computed using ${n}_{0i}$ and ${N}_{i}$ as: ${n}_{i}=\frac{{n}_{0i}*{N}_{i}}{{n}_{oi}+{N}_{i}-1}$, $i=\mathrm{1,2}$ denoting the sample of health center size or hospital size determined. Thus, the sample size for each stratum is ${n}_{Health centers}=552$ ${n}_{Hospitals}=77$ And the total sample size is computed as $n={n}_{Health centers}+{n}_{Hospitals}=629$

The final sample size represents 15% of health centers and 30% of hospitals. Once the total sample was determined, sample allocation was made based on the probability proportional to their size (PPS) for each region using the $\frac{n}{N}=\frac{{n}_{r}}{{N}_{r}}$, formula. Then the sample allocated for each region was computed with ${n}_{r}=\frac{n}{N}*{N}_{r}$.

During the computation of required samples for regions, the sample size for Dire Dawa and Harari regions for hospitals was equivalent to zero but rounded up to 1 to ensure representation of the regional hospitals, and the final sample size reached 631.

### Sampling strategy

Multistage sampling techniques were used to select health facilities for the study. Available health facilities in the nation were considered during the selection process, and the final list of health facilities for the study was selected using the lottery method. Then one health post under the chosen health center is included in the study.

#### Survey implementation status of MDSR system evaluation

**Fig. 2:**
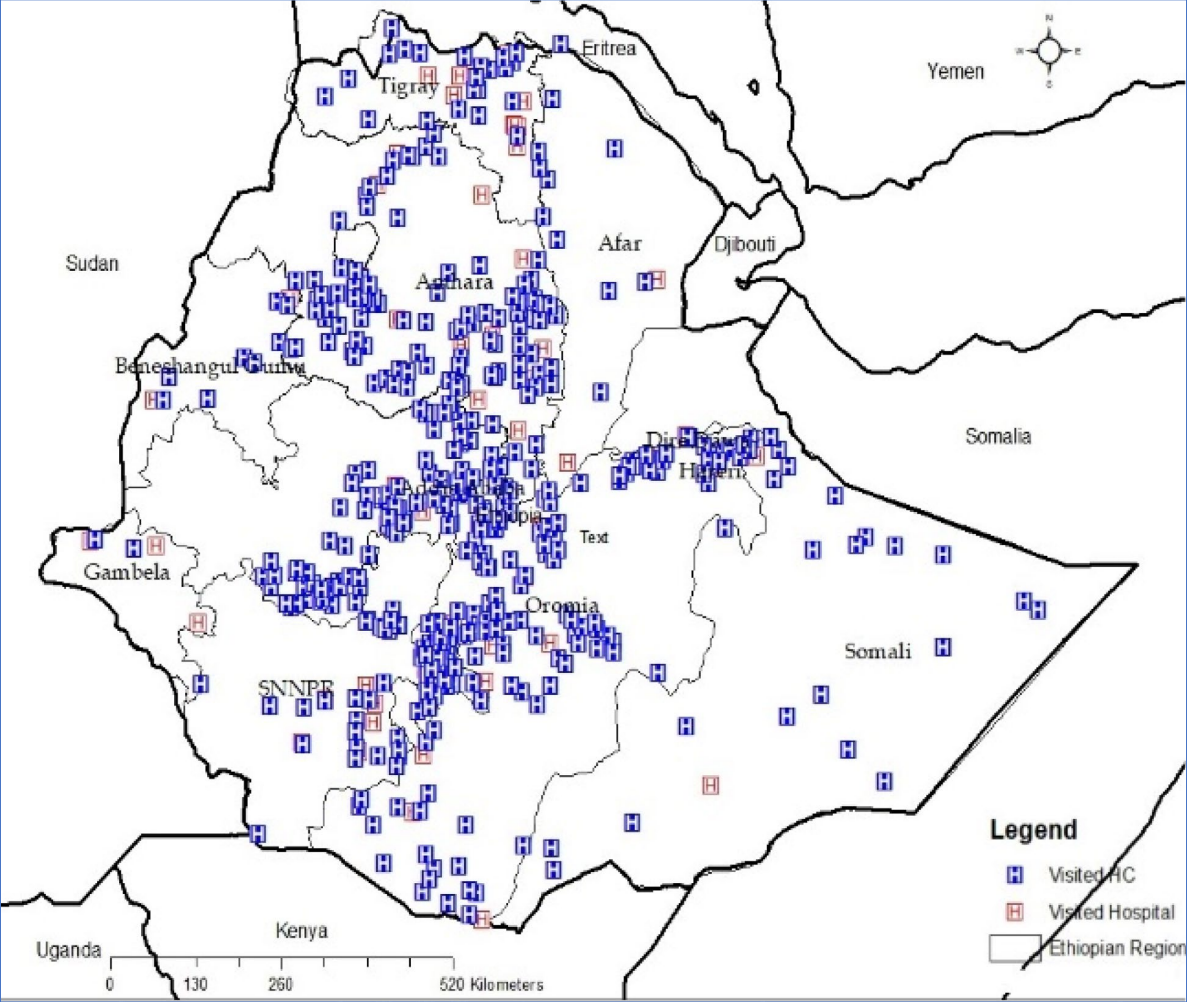
Geographical Distribution of Visited Health Facilities by Type and Region, National MDSR System Evaluation, Ethiopia, 2020

Among the health facilities selected for this assessment during the planning phase, data collection was conducted at 445 (80.5%) health centers, 74 (94.9%) hospitals, and 369 (68.5%) health posts. Due to conflict and inaccessibility, some health facilities were not visited during the survey. The geographical distribution of visited health facilities is indicated in the figure below (Fig. 2).

#### Survey respondents

The required data were collected from a different mix of professionals, experts, and officials working at visited health facilities and health posts. About 340 (65.5%) of respondents were facility surveillance officers, followed by 98 (18.8%) Chief Executive Officers (CEOs) and medical directors of health facilities. A summary of the survey implementation status and background characteristics of survey respondents is indicated below (Table 1).

**Table 1:** Survey implementation status at health facilities and health posts, and background of the interviewees, National MDSR system evaluation, Ethiopia, 2020

**Facility type**	**Categories**	**Tigray**	**Afar**	**Amhara**	**Oromia**	**Somali**	**Benishangul Gum**	**SNNPR**	**Gambella**	**Harari**	**Addis Ababa**	**Dire Dawa**	**National**
**Health Centers**	**Available**	273	78	849	1317	165	40	731	32	8	97	15	3545
	**Sampled (N)**	29	14	118	236	27	7	100	5	1	14	2	553
	**Visited (n)**	24	11	99	203	23	8	56	3	1	14	3	445
	**Percentage**	82.8	79.0	83.9	86	85.2	114.3	56	60.0	100	100	150	80.5
**Hospitals**	**Available**	31	6	73	101	10	6	28	3	1	13	2	274
	**Sampled (N)**	9	2	20	18	3	2	17	2	1	3	1	78
	**Visited (n)**	10	2	18	15	3	2	16	3	1	3	1	74
	**Percentage**	111.1	100	90.0	83.3	100	100	94.1	150	100	100	100	94.9
**Health Posts**	**Planned (n)**	29	14	118	236	27	7	100	5	1	NA	2	539
	**Visited (n)**	12	10	97	165	21	8	53	2	1	NA	0	369
	**Percentage**	50.0	71.0	82.2	69.9	77.8	114.3	53.0	40.0	100	NA	0.0	68.5

### Data collection tools, quality management, and evidence generation

Standardized quantitative assessment tools designed using ODK with integrated data quality assurance features using android-based mobile tablets were used for data collection. The refinement of data collection tools and procedures was done based on observations from field-level pre-testing. Field epidemiology residents mobilized from universities, national MDSR Technical Working Group (TWG) members, and MDSR officers from regional health bureaus were participated during the data collection and as field team coordinators.

All data collectors were trained for three days on general data collection guidelines and the basic components of the Maternal Death Surveillance and Response System. Each data collection team's implementation was tracked by the central data manager, who also assigned a central survey coordinator. Field supervisors verified the collected data for accuracy and completeness at the field level and then transferred it to a secure central server in the national PHEM data management center.

Data cleaning was carried out during and after the completion of the data collection time and before the final data analysis and information generation process. Data were exported to Stata 14, and descriptive statistics were disaggregated by type of facility.

## Findings

### MDSR system implementation status

**Fig. 3:**
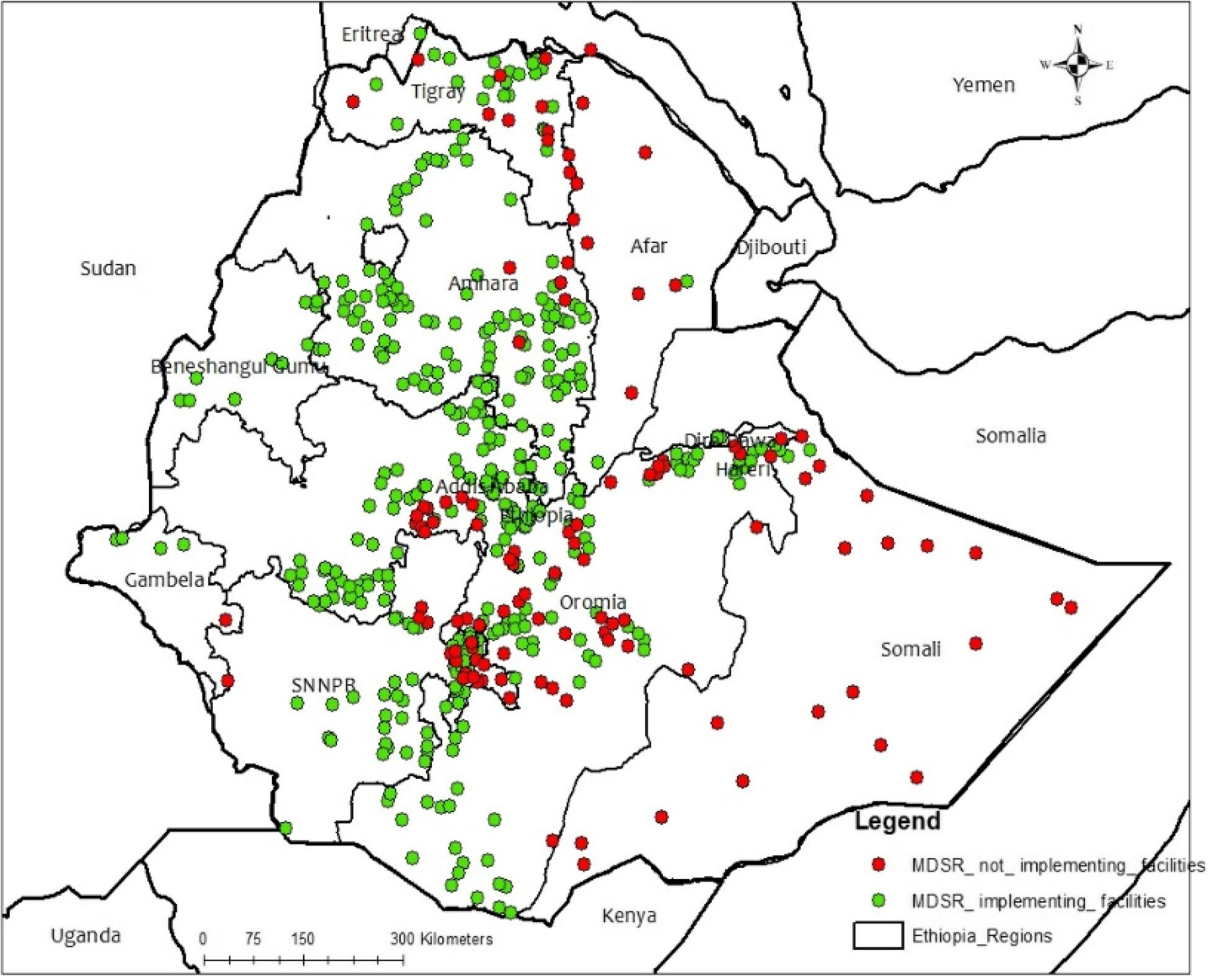
Geographical distribution of MDSR system implementing health facilities by region, National MDSR system evaluation, Ethiopia, 2020

A total of 400 (77.1%) visited health facilities implementing the MDSR system during the visit. Of all implementing health facilities, 332 (74.6%) and 68 (91.9%) were health centers and hospitals respectively. Furthermore, a total of 264 (71.5%) health posts were implementing the MDSR system during the time of the visit. The geographical distribution of the MDSR system implementing health facilities shows significant variation among different regions in the nation. System implementation at health facilities in the Somali and Afar regions is low. A substantial difference in community-level MDSR system implementation status was also observed among regions in the nation (Table 2, Fig. 3).

**Table 2:** MDSR system implementation status and year of implementation by type of health facilities, National MDSR system evaluation, Ethiopia, 2020

**Health Facility level MDSR system implementation**	**Variables**	**Categories**	**Total**	**Percent (%)**
	MDSR system implementing health facilities out of 519 visited health facilities		400	77.1
	MDSR implementing health facilities with assigned MDSR focal person		279	69.8
	MDSR implementing facilities with trained MDSR focal person in < = 2 years		153	54.6
	MDSR system implementation by type of health facilities	Health Center	332	74.6
		General Hospital	29	87.9
		Primary Hospital	25	92.6
		Referral Hospital	14	100
	Years of MDSR system implementation	Less than 1 year	123	30.8
		1 to 3 years	39	9.8
		Greater than or equal to 3 years	238	59.5
**MDSR system implementation at a community level**	Implementing health posts out of 369 visited health posts		264	71.5
	Trained HEWs with the updated IRT(*n* = 264)		101	38.5
	HEWs trained/get on-job orientation on MDSR (*n* = 264) – less or equal to 2 years		40	56.3
	HEWs trained/get on-job orientation on MDSR (*n* = 264) – less or equal to 2 years		31	43.7
	Years of MDSR system implementation	Less than 1 year	17	6.4
		1 to 3 years	89	33.6
		Greater than or equal to 3 years	159	60.0

Based on this evaluation findings, 279 (69.8%) of MDSR-implementing health facilities have assigned MDSR focal persons to coordinate MDSR-related activities in their institutions. Out of the assigned experts at health facilities, 153 (54.6%) were trained on the MDSR system at least once in the last 24 months. Additionally, 101 (38.5%) HEWs reported that they are trained on MDSR, and out of the trained, 40 (56.3%) received on-the-job orientation or training on community-level MDSR systems in the last 24 months.

### Identification/detection and notification of maternal deaths

Of the total MDSR system-implementing health facilities, 162 (40.5%) and 169 (42.3%) had case definitions and maternal death notification formats, respectively. Besides, experts are assigned to routinely review registration books and client charts for unreported maternal deaths at 224 (56.0%) facilities. Furthermore, about three-fourths (75.5%) of MDSR implementing facilities (both health centers and hospitals) and 314 (94.6%) health centers reported the existence of established mechanisms for identification and formal notification of maternal deaths occurring in their health facilities and from their community respectively (Table 3).

**Table 3:** Indicators for Identification and Notification of Maternal Death and Death Registration, Archiving, and Reporting Practice at health facilities and health posts, national MDSR system evaluation, Ethiopia, 2020

**Identification and Notification of Maternal Deaths**	**Health Facility Level MDSR system**	**Variables**	**Categories**	**Total**	**Percent (%)**
		Number of MDSR implementing health facilities		400	
		Availability of case definition (*n* = 400)		162	40.5
		Availability of notification format (*n* = 400)		169	42.3
		Mechanism to receiving MD at HCs (*n* = 400)		302	75.5
		Availability of experts to review Charts (*n* = 400)		224	56.0
		Overall readiness of health center for I&N (*n* = 332)		37	11.6
		Availability of established mechanism for receiving death from the community (*n *= 332)		314	94.6
		Overall readiness of hospitals for Identification & notification of maternal deaths (*n* = 68)		14	20.6
	**Community-Level MDSR system**	Number of MDSR Implementing health posts		264	
		Health posts with case definition—locally translated		39	14.8
		The availability of a mechanism for receiving notification from communities = Yes		231	87.5
		Means of receiving MD from the community(*n* = 264)	By HDA	205	88.7
			Direct from community	155	67.1
		Screening of WRA (women of reproductive age)		203	76.9
		Methods of maternal death identification and notification (*n* = 204)	House visit	185	91.1
			During meeting	89	43.8
			Notification from any community member	115	56.2
**Maternal Death Registration, Archiving, and Reporting Practice**	**Health Facility Level MDSR system**	Archiving practice of maternal D report		301	75.1
		Communication means available		390	97.5
		PHEM weekly reporting format		330	83.5
		Rumor logbook		124	31.4
		Registration book		282	71.4
		MDRF format		192	48.6
	**Community-Level MDSR system**	Revised PHEM format (*N* = 264)		208	78.8
		Rumor Logbook		73	27.7
		Registration book (*N* = 264)		212	80.0
		Communication means		245	92.0
		Types of communication available for MD in HP	Paper-based	62	25.2
			Phone call	17	6.9
			Text message	1	0.4
			Paper and phone	137	55.0
			All means	28	11.4

Of the total implementing health facilities, only 37 (11.6%) and 14 (20.6%) MDSR-implementing health centers and hospitals have optimal readiness for conducting maternal death identification and notification process. Among system-implementing health posts, 39 (14.0%) have locally translated community case definitions for maternal death. The vast majority, 231 (87.5%), of the health posts reported the availability of established mechanisms for receiving maternal death reports from the community. Besides, a total of 203 (76.9%) MDSR implementing health posts reported the existence of an established system to screen death of women of reproductive age group occurred in their catchment community (Table 3).

### Maternal death registration, archiving, and reporting practice

301 (75.1%) and 390 (97.5%) implementing health facilities that archive maternal death reports and have a means of communication for maternal death data reporting to the next level, respectively. Regional variation is also observed in archiving maternal death reports. None of the MDSR system-implementing health facilities in the Afar region had archived maternal death reports during the visit (Table 3).

### Maternal death investigation, review, and evidence generation

268 (67.0%) MDSR-implementing health facilities (both health centers and hospitals) assigned experts to oversee the investigation of suspected maternal deaths in their institution, and 158 (39.5%) health centers assigned responsible experts (surveillance focal person) to monitor the investigation status of reported maternal deaths by HEWs from their catchment areas. Additionally, 349 (87.2%) health facilities (both health centers and hospitals) had a maternal death review committee in their respective institutions. Only 42 (12.4%) of the review committee held more than or equal to six death review coordination meetings during 2011 EFY (2019 GC). Among them, 275 (78.7%) are chaired by CEOs or medical directors, followed by surveillance focal persons 54 (15.3%). In addition, only 6 (1.5%) health facility respondents claimed that they were engaged in routine analysis and information generation of maternal death data in their health facilities at least once.

Furthermore, only 26 (6.5%) and 45 (11.3%) facilities reported the availability of finance for MDSR system implementation and functional computers for surveillance in their health facility, which varied across regions. Of all the MDSR system-implementing health facilities, 23 (6.9%) health centers and 18 (26.5%) hospitals have optimal readiness to perform the overall maternal death investigation and review process at the national level. Regarding community-level maternal death investigation status, only 28 (10.6%) health posts had locally translated verbal autopsy format to investigate maternal deaths (Table 4).

**Table 4:** Summary of indicators for maternal death investigation and review practice, national MDSR system evaluation, Ethiopia, 2020

**Health facility level MDSR system—Maternal Death Investigation and confirmation**	**Variables**	**Categories**	**National**	**Percent (%)**
	Total MDSR system implementing health facilities		400	
	Availability of experts to investigate suspected MD in HF (*n* = 400)		268	67.0
	Responsible expert to monitor the investigation of reported deaths = Yes		158	39.5
	Availability of MDSR review committee for confirmation (*n* = 400)		349	87.3
	MDSR review committees which held > = six death review meetings—2011 EFY (2019 G.C)		42	12.4
	MDSR review committees which held < six death review meetings—2011 EFY (2019 G.C)		296	87.6
	Availability of facility-based abstraction format(*n* = 400)		117	29.6
	Overall HC readiness for investigation and confirmation *N* = 332		23	6.9
	Overall hospitals readiness for Case investigation and confirmation(*n* = 68)		18	26.5
	Available finance for MDSR system implementation		26	6.5
	Availability of functional computer for MDSR system		45	11.3
**Health post-level MDSR system—Maternal Death Investigation and confirmation**	Total number of MDSR system Implementing health posts		264	
	Availability of locally translated Verbal Autopsy (VA) (*N* = 264)		28	10.6
	Who is responsible for investigating MDs	Catchment HF focal (*N* = 265)	96	36.4
		Midwives	76	28.8
		RRT (*N* = 265)	94	35.6
		HEW (*N* = 265)	150	56.0

## Discussion

Having a higher geographical coverage of a system or evidence that shows an optimal proportion of implementing health system structures is considered a proxy indicator for generating evidence about the disease or event under surveillance. These indicators can provide valuable information about the status of a surveillance system's implementation status by being a measurement tool during monitoring and evaluation [3, 12, 16].

According to national PHEM guidance, at least 80% of health facilities are expected to implement the MDSR system to have a complete picture of the country's maternal mortality burden and surrounding factors. However, this system evaluation found that 77.1% of health facilities (74.6% for health centers and 91.9% for hospitals) and 71.5% of visited health posts are implementing the system, which is lower than the expected national standard. This may be related to the lower occurrence of maternal deaths at health centers than hospitals and not considering deaths happening in their catchment community as their responsibilities. Furthermore, there is a tendency to send near-death mothers to catchment hospitals as a referral. Despite being lower than the expected national level standard, study findings from Guinea community-based maternal deaths surveillance, this study figure was also higher than the findings from a baseline survey conducted in 2015 to assess the status of death review implementation in low- and middle-income countries [17–19].

This study finding also reveals that the geographical distribution of MDSR system-implementing health facilities in the country varies significantly across different regional structures. The lack of MDSR training or orientation for HEWs and community health agents, a lack of locally translated materials like case definitions for maternal death, a financial shortage, and other factors in emerging regions could contribute to this lower implementation of the system in the area [20–22]. In addition to the sociocultural and religious factors which can significantly influence maternal death reporting, investigation, and reporting in the areas, the absence of a customized system for primary health care delivery in emerging regions may also significantly influence the MDSR system implementation in emerging regions.

Furthermore, low levels of community engagement in emerging regions can significantly contribute to this. Our argument is supported by a study finding conducted in the central part of Malawi and the eastern parts of Ethiopia, demonstrating that low levels of community engagement or participation in the MPDSR process are system implementation barriers [23].

The availability and functionality of the MDSR committee at a given health facility is a critical platform for monitoring system implementation status and ensuring the system can prevent similar deaths in the future field [5, 12]. The findings of this evaluation show that more than three-quarters of the visited health facilities had an MDSR committee. Still, only one-tenth of the facilities had a functional MDSR committee that held more than six death review coordination meetings per year. This finding demonstrates a significant gap in the availability and functionality of the required emergency coordination platforms at health facility levels, which is lower than the findings of previous studies in Guinea and Malawi [19, 24]. It could be due to a lack of support and attention received from respective institution leaders and a lack of involvement of concerned stakeholders.

According to this assessment, lower than half of the visited health facilities had case definitions and maternal death notification formats. Furthermore, about 45.0% of MDSR-implementing health facilities had no assigned surveillance focal person and experts to conduct routine reviews of registration books and client charts to identify maternal deaths that are not reported in their health institutions. This could be attributed to the internal rotation of trained staff within the facility, staff turnover, and low budget allocation for capacity-building activities for MDSR. The failure to designate a focal point may also reflect the failure of respective facility managers in this aspect.

Considering that the majority (75.0%) of maternal deaths are expected to happen at the community level, the shortage of locally translated case definitions for maternal death and lack of training on the implementation of MDSR at the community level can significantly reduce the detection and notification of maternal deaths at the community level. This could dramatically impact the functionality of the community-level MDSR system implementation [25–28]. Locally translated surveillance materials are friendlier and easier to understand, potentially improving quality data reporting and motivation of community health workers. The lower-level readiness of health facilities and health posts evidenced in this finding could be related to the system's ten percent implementation status after seven years in the national [29].

Because the maternal death review, analysis, and response elements cannot function without them, recording and documentation are essential components of MDSR. According to the national technical guideline for MPDSR, all health facilities should record, document, or archive all notified or reported deaths [12]. According to this assessment result, approximately three-fourths of implementing health facilities archived maternal death reports.

Most of the MDSR implementing health facilities and health posts visited use a hard copy for service delivery registry and a combination of paper and phone (55.9%), with a paper-based one-fourth for information communication. Our findings contrast with another study conducted in Guinee, which discovered that all visited health facilities use electronic data communication and have maternal death databases [24]. This could be due to the lower level of attention given by the nation to the issue and a lack of funds and infrastructure compared to the indicated country.

This study also reveals that the overall readiness of health facilities for investigation and review varies across different regions in the nation. The disparities in detecting and notifying maternal deaths across regions may be due to a lack of trained human resources, training, guidelines and formats, work overload, and healthcare workers' negative attitudes toward maternal death detection and notification.

## Conclusions and recommendations

### Conclusions

This system evaluation profiled the MDSR system performance status at the health facility and community level across all regions and city administrations of Ethiopia. The evidence indicated that the system is not well-equipped to handle the very objective of the surveillance, which was to eliminate preventable maternal death by obtaining and using the information on each maternal death to guide public health action. Even though there may be a long list of surrounding factors for this suboptimal achievement, the lower-level-level commitment of experts assigned to coordinate MDSR-related activities at the health facilities and health facilities' leadership commitment takes the greater portion. Additionally, findings on the community-level MDSR system implementation status uncover a gap in community MDSR systems in capturing and investigating community death. Even though most of the implementing health facilities established a committee in their respective institutions, a considerable number of the committees were not functional, as evidenced by the absence of routinely scheduled meetings to discuss maternal death as per the guidance. Moreover, routine data analysis was low, violating the surveillance's goal and cannot proceed beyond counting deaths.

### Recommendations

Even though this system evaluation shows the national MDSR is not working as expected, this system evaluation showed that the national-level MDSR system implementation in Ethiopia is partially satisfactory and encouraging. However, gaps remain, and upgrading the current system offers a unique opportunity to implement the necessary structural changes and exploit the system to its potential.

Specific recommendations categorized under WHO building blocks are provided below.

#### Leadership and Governance

- Establishing a system to monitor emergency coordination platforms' availability and functionality at all levels.
- Considering the system as a "flagship" program to foster the implementation, monitoring, and evaluation at the health facility and community level.

#### Workforce

- Establish a system for routine capacity building, refreshment training, and gap filling for the assigned PHEM officers and review committee members.
- Ensure well integration of MDSR and other surveillance systems in PHEM at health facilities.

#### Healthcare financing

- Ensure sustainable financial support for the system implementation, strengthening, monitoring, and evaluation.

#### Health Information System for MDSR

- Establish well-organized data management system for the MDSR integrated with the existing national PHEM.
- Digitalization for real-time data collection, evidence generation, and sharing.

### Service delivery

- The system should be designed to fit emerging regions' health service delivery to address the implementation gap and monitoring & evaluation at emerging regions.

#### Logistics and supply

- System should be designed to avail the required resources for maternal death identification, reporting, information generation, and sustainability sharing.
- Avail computer, internet service, pocket guide for health extension workers, and health facility level MDSR review committee members.

## Data Availability

The datasets used for this study are available from the corresponding author upon reasonable request.

## Abbreviations

HFs: Health Facilities
HPs: Health Posts
MCH: Maternal and Child Health
MDSR: Maternal Death Surveillance and Response
MMR: Maternal Mortality Rate
ODK: Open Data Kit
PHEM: Public Health Emergency Management
PHEs: Public Health Emergencies
SDG: Sustainable Development Goals
SERO: Scientific and Ethical Research office
SNNPR: South Nation Nationality and Peoples Region
TWG: Technical Working Group
UNFPA: United Nations Population Fund
UNICEF: United Nations International Children's Emergency Fund
WHO: World Health Organization
